# Raman Microspectroscopy Identifies Biochemical Activation Fingerprints in THP-1- and PBMC-Derived Macrophages

**DOI:** 10.3390/biomedicines10050989

**Published:** 2022-04-25

**Authors:** Nora Feuerer, Daniel A. Carvajal Berrio, Florian Billing, Sören Segan, Martin Weiss, Ulrich Rothbauer, Julia Marzi, Katja Schenke-Layland

**Affiliations:** 1 Institute of Biomedical Engineering, Department for Medical Technologies and Regenerative Medicine, Eberhard Karls University Tübingen, 72076 Tübingen, Germany; nora.feuerer@nmi.de (N.F.); daniel.carvajal-berrio@uni-tuebingen.de (D.A.C.B.); katja.schenke-layland@uni-tuebingen.de (K.S.-L.); 2 NMI Natural and Medical Sciences Institute at the University of Tübingen, 72770 Reutlingen, Germany; florian.billing@nmi.de (F.B.); soerensegan@gmail.com (S.S.); martin.weiss@med.uni-tuebingen.de (M.W.); ulrich.rothbauer@nmi.de (U.R.); 3 Cluster of Excellence iFIT (EXC 2180) “Image-Guided and Functionally Instructed Tumor Therapies”, Eberhard Karls University Tübingen, 72076 Tübingen, Germany; 4 Department of Women’s Health, Research Institute of Women’s Health, Eberhard Karls University Tübingen, 72076 Tübingen, Germany; 5 Pharmaceutical Biotechnology, Eberhard Karls University Tübingen, 72076 Tübingen, Germany; 6 Department of Medicine/Cardiology, University of California Los Angeles (UCLA), Los Angeles, CA 90095, USA

**Keywords:** macrophage polarization, Raman imaging, molecular phenotyping, immune in vitro test system

## Abstract

(1) The monocytic leukemia cell line THP-1 and primary monocyte-derived macrophages (MDMs) are popular in vitro model systems to study human innate immunity, wound healing, and tissue regeneration. However, both cell types differ significantly in their origin and response to activation stimuli. (2) Resting THP-1 and MDMs were stimulated with lipopolysaccharide (LPS) and interferon γ (IFNγ) and analyzed by Raman microspectroscopy (RM) before and 48 h after activation. Raman data were subsequently analyzed using principal component analysis. (3) We were able to resolve and analyze the spatial distribution and molecular composition of proteins, nucleic acids, and lipids in resting and activated THP-1 and MDMs. Our findings reveal that proinflammatory activation-induced significant spectral alterations at protein and phospholipid levels in THP-1. In MDMs, we identified that nucleic acid and non-membrane-associated intracellular lipid composition were also affected. (4) Our results show that it is crucial to carefully choose the right cell type for an in vitro model as the nature of the cells itself may impact immune cell polarization or activation results. Moreover, we demonstrated that RM is a sensitive tool for investigating cell-specific responses to activation stimuli and monitoring molecular changes in subcellular structures.

## 1. Introduction

Macrophages were thought to be merely phagocytic cells that clear tissues of pathogens and cellular debris [1]. However, macrophages are highly heterogeneous and versatile cells, which can adapt to a vast range of different phenotypes, often termed polarization, and take over distinct tasks in human immunity. For example, macrophages act as antigen-presenting cells that activate the adaptive immune system and trigger inflammation, secrete and remodel components of the extracellular matrix, and they interact with non-immune cells such as fibroblast and endothelial cells. Therefore, macrophages play a profound role in fibrosis and wound healing [2,3,4]. Macrophage plasticity is primarily manifested in comprehensive transcriptional and metabolic alterations that are precisely regulated by a variety of signaling pathways, leading to extensive changes in the biochemical composition of the cells [5,6].

RM and Raman imaging have become important methods in materials and biological sciences to analyze the biochemical composition of inorganic and organic samples [7,8]. In biomedicine, Raman-based methods are increasingly used for live-cell studies. They do not require sample pre-processing such as fixation or antibody staining, allowing the study of living specimens [9,10]. Because the chemical composition of a cell is complex and highly heterogeneous, the evaluation of the Raman spectrum of a cell requires intense data processing and careful interpretation. However, modern algorithms already allow the identification and visualization of subcellular structures such as DNA, lipids, or proteins based on their Raman fingerprint [9,11]. Moreover, identification of minute changes in the spectral signatures can be assigned to changes in the molecular composition of a cell or a cellular structure and therefore allow for marker-independent cellular phenotyping [12,13] or monitoring of (patho-)physiological changes or drug-induced effects [14,15].

The THP-1 monocytic cell line and MDMs circulating in the peripheral blood are both popular models for studying macrophage biology [16]. Both cell models come with the typical advantages and disadvantages of cell lines in contrast to primary cells: easy access, safety concerns, and reproducibility must be carefully balanced when choosing one over the other. In addition, there is ample evidence that the malignant origin of the THP-1 cell line causes severe alterations in cell biology and activation [17]. The capability of THP-1 cells to adequately mimic macrophage biology has therefore been questioned on numerous accounts [18]. 

This study is designed to implement Raman imaging and multivariate data analysis to holistically analyze and compare cellular phenotypes of THP-1 cells and MDMs on a subcellular resolution. On a single cell level, insights into the biochemical remodeling of nucleic acids, lipids, and cytoplasmic structures through cell activation processes and differences in activation patterns will be elaborated.

## 2. Materials and Methods

### 2.1. Peripheral Blood Mononuclear Cell-Derived Monocyte Isolation and Culture

Human blood samples were retrieved in accordance with the Declaration of Helsinki, which was approved by the Ethics Committee of the Medical Faculty at the University of Tübingen (IRB# 495/2018BO2). Peripheral blood mononuclear cells (PBMCs) were isolated using density gradient centrifugation of freshly collected whole blood from one healthy volunteer after obtaining informed consent [19]. Monocytes were isolated by plastic adherence as described before [20]. After isolation, PBMCs were seeded at a concentration of 5 × 10^6^ cells/cm^2^ at 37 °C and 5% CO_2_ in a culture medium. After 2 h, all non-adherent cells were aspirated and removed. The remaining adherent monocytes were washed with PBS and continually cultured to mature into macrophages.

### 2.2. Macrophage Maturation and Polarization

RPMI 1640 with Glutamax™ supplemented with 100 μg/mL streptomycin, 100 U/mL penicillin, and 10% heat-inactivated fetal bovine serum (FBS) (all from Thermo Fisher, Waltham, MA, USA) was used to culture monocytes in polystyrene 24-well cell culture plates (ibidi, Planegg, Germany) at 37 °C and under a 5% CO_2_ humidified atmosphere.). The culture medium was supplemented with 50 ng/ mL macrophage colony-stimulating factor (M-CSF; Biolegend, Amsterdam, The Netherlands) to mature monocytes into macrophages over 8 days. Medium changes were performed on days 3 and 5. On day 5, 100 ng/mL Lipopolysaccharide (LPS) (*E. Coli*, O111:B4; Merck Millipore, Darmstadt, Germany) and 100 ng/mL IFNγ (Biolegend) were added to induce macrophage activation. Control M0 (resting) macrophages were not stimulated, but the medium was changed on days 3 and 5. Four hours prior to analysis, MDMs were treated with GolgiPlug™ (BD Bioscience, NJ, USA) to block the intracellular protein transport and enhance the detectability of cytokine production. 

### 2.3. THP-1 Culture

The human monocytic leukemia cell line THP-1 (ATCC, Manassas, VA, USA) was cultured in T75 flasks (Greiner Bio-One, Kremsmünster, Austria) in RPMI 1640 with Glutamax™ supplemented with 100 μg/mL streptomycin, 100 U/mL penicillin and 10% FBS at 37 °C, under a 5% CO_2_ humidified atmosphere. Cells were split upon a concentration confluency between 8 × 10^5^ and 1 × 10^6^ cells/ ml. For all experiments, cells from passages 10 to 15 were used. THP-1 cells were treated with 50 ng/mL phorbol-12-myristate-13-acetate (PMA) (Sigma-Aldrich, St. Louis, MO, USA) for 48 h to induce macrophage differentiation. The differentiation process was performed in 6-well plates (Corning, Wiesbaden, Germany) at a density of 2 × 10^6^ cells/ well. Subsequently, after differentiation, the PMA-containing medium was removed, and cells were cultured for an additional 48 h without PMA. PMA-treated cells were detached using 0.05% trypsin/EDTA (Gibco) and reseeded in a polystyrene 24-well cell culture plate (ibidi GmbH) for 48 h. For activation, cells were stimulated 2 h after seeding with 100 ng/mL LPS and 100 ng/mL IFNγ for 48 h. 

### 2.4. Flow Cytometry

For flow cytometry (FC) analysis, cells were dissociated with Accutase (Biolegend) and processed as described before [20]. For extracellular antigen staining, the following antibodies were used: CD86-Pacific Blue™, human leukocyte antigen DR (HLA-DR)-Brilliant Violet (BV) 510, CD206-FITC, and CD163-PE/Cy7 (all from Biolegend). For intracellular cytokine staining, cells were resuspended in Cytofix/ Cytoperm solution for 20 min at 4 °C and washed with Permwash (both BD Bioscience, East Rutherford, NJ, USA). The following intracellular antibodies were then diluted in Permwash at a concentration of 1:50, and cells were stained for 30 min at 4 °C in the dark: tumor necrosis factor α (TNFα)-BV711 (#502939, Interleukin 6 (IL-6)-PE/Cy.5 (#501117), Interleukin 10 (IL-10)-PE/Dazzle (#501425), Monocyte chemoattractant protein 1 (MCP-1)-PE/Cy7 (502613) (all Biolegend) and Interleukin 1 Receptor Antagonist (IL1RA)-PE (#340525, BD Bioscience). Mean fluorescence intensities (MFI) were analyzed using a BD Biosciences LSRFortessa Cytometer (East Rutherford, NJ, USA). Data were interpreted using FlowJo™ 10.4.2 (Tree Star, Ashland, OR, USA). A forward scatter versus sideward scatter gating strategy was employed to exclude debris, doublets, and dead cells (7-AAD staining, Biolegend). Each experiment was compensated using UltraComp eBeads (Thermo Fisher, Waltham, MA, USA) and software-based automatic compensation to correct fluorescence spillover. Fluorescence minus one controls were performed to determine the background fluorescence of the antibody panel. 

### 2.5. Raman Microspectroscopy

RM was performed using a customized WiTec alpha 300R Raman system (WiTec GmbH, Ulm, Germany) equipped with a green laser (532 nm) and a charged-coupled device spectrograph with a grating of 600 g/mm. Raman images comprising the whole cell area were acquired. 30 × 30 µm scans of single macrophages were acquired using a 63× apochromat water dipping objective (Carl Zeiss AG, Jena, Germany), an integration time of 0.5 s, a pixel resolution of 1 × 1 µm, and a laser power of 50 mW. A total of 30 cells were measured for each M0 and M1 replicate.

### 2.6. Data Processing

Raman data were processed using the Project FIVE 5.2 software (WITec GmbH, Ulm, Germany). Cosmic rays were removed, and a baseline correction was employed on all spectra. Spectra were cropped to the range from 300 cm^−1^ to 3045 cm^−1^. True Component Analysis (TCA) was employed to identify major spectral components in the images. To extract single spectra, masks were generated based on TCA heat maps. To reduce the dimensionality of the spectral data, principal component analysis (PCA) was performed using the Unscrambler × 14.0 software (Camo Software, Oslo, Norway) [21]. For PCA analysis, the spectral fingerprint region between 400–1800 cm^−1^ was investigated. PCA results are presented as score plots and loading plots. In brief, PCA is an unsupervised algorithm elaborating spectral differences and similarities on a vector-based approach. The identified vectors are also called principal components (PCs), where PC-1 explains the most relevant difference in the data set, PC-2 the second most relevant difference, etc. PC-1 often only describes differences based on spectral intensities or background signals. PCs were selected based on a clustering of the data sets of interest and the biological relevance of the most influencing peaks in the assigned loadings plot. 

### 2.7. Statistical Analysis

All statistical analyses were performed by a two-tailed student’s t-test with a *p*-value of 0.05 with Graph Pad Prism 9.2.0 (Graph Pad Software Inc. San Diego, CA, USA). Graphs are box and whiskers plots where the whiskers have been calculated using the Tukey method. All experiments were performed as triplicates. MDMs originate from 3 different isolations from one donor. A total of 90 cells per condition were measured. 

## 3. Results

### 3.1. Definition of Activation Patterns of THP-1 Macrophages and MDMs by Flow Cytometry

**Figure 1:**
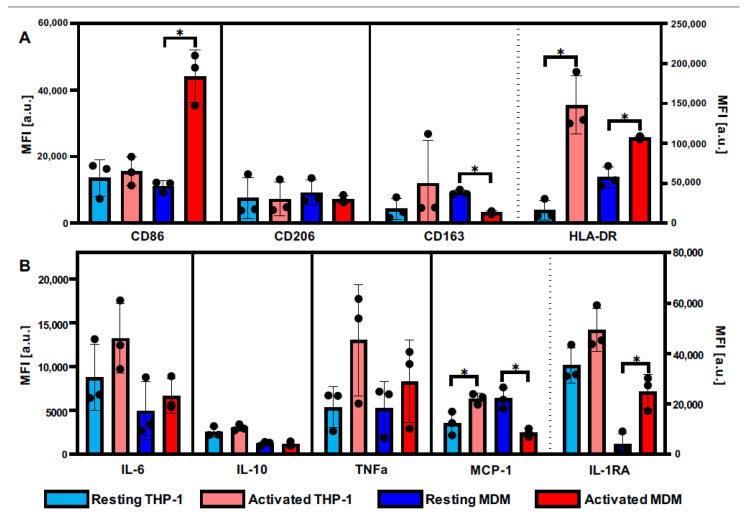
THP-1-derived macrophages and MDMs differ in their molecular activation patterns. Expression of (**A**) surface antigens and (**B**) cytokines of THP-1 (light colors) and MDMs (intense colors) before (blue) and after (red) LPS-IFNγ activation. Data represent mean fluorescence intensity (MFI) values ± SD; *n* = 3; pairwise comparison via student’s *t*-test, * *p* ≤ 0.05. Boxplots after the dotted line are assigned to the right y-axis. HLA-DR: human leukocyte antigen DR isotypes; IL: interleukin; TNF: tumor necrosis factor; MCP: monocyte chemoattractant protein; IL-1RA: interleukin 1 receptor antagonist.

State-of-the-art characterization of immune cells and their subpopulations includes investigating their expression of specific surface markers and cytokines. To define macrophage activation by LPS and IFNγ, routine FC was performed (Figure 1). Upon LPS-IFNγ activation, THP-1 and MDMs responded with a significantly increased expression of the surface antigen HLA-DR and elevated expression patterns of cytokines IL-6, TNF, and IL-1RA, which indicate an activated cellular phenotype. Similarly, no significant changes in CD206 and IL-10 were observed for both cell types. Expression baselines and activation levels differed between THP-1 and MDMs for several of these markers. Differences between THP-1 and MDMs were also reflected in increased CD86 levels as well as decreased CD163 levels in activated macrophages, solely shown in MDMs, but not in THP-1. For MCP-1, inverse effects were observed between the two groups.

### 3.2. Raman Imaging Provides Spatial Resolution of Subcellular Structures in Macrophages

**Figure 2:**
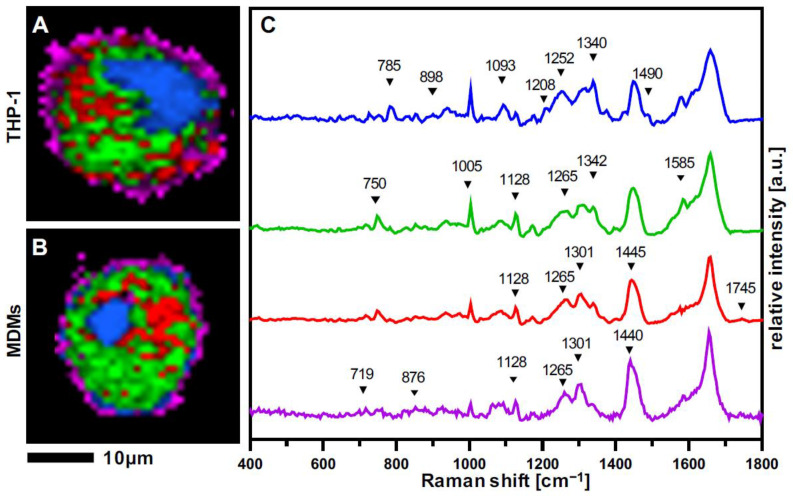
Raman imaging of resting macrophages. True component analysis (TCA) segmentation of subcellular structures allowed to generate intensity distribution heatmaps of (**A**) THP-1 macrophages and (**B**) MDMs. Characteristic spectral signatures (**C**) identified and localized nucleic acids (blue), cytoplasm (green), lipids A (red), and lipids B (purple). Scale bar equals 10 µm.

THP-1 and PBMC-derived monocytes were matured into M0 and M1 macrophages and characterized by RM and Raman imaging. Raman scans were segmented by TCA into false color-coded intensity distribution heat maps (Figure 2A,B). Four major spectral components were identified, localizing nucleic acids (blue), cytoplasm (green), and two lipid-related components—referred to as *lipids A* (red) and *lipids B* (pink) (Figure 2C).

Nucleic acids were identified based on characteristic bands at 785 cm^−1^, 1093 cm^−1^, 1340 cm^−1,^ and 1576 cm^−1^, assigned to DNA. The spectral component describing cytoplasmic molecules displays bands at 750 cm^−1^, 1005 cm^−1^, 1585 cm^−1,^ and 1660 cm^−1^ associated with molecular vibrations from proteins and mitochondrial activity. The lipid feature was further distinguished into *lipids A*, rather located inside the cells with representative Raman bands at 1128 cm^−1^, 1265 cm^−1^, 1301 cm^−1,^ and 1445 cm^−1^, as well as *lipids B*, which showed additional bands at 719 cm^−1^ and 876 cm^−1^ located at the outer surrounding of the cell. The same major spectral components and similar distribution patterns were retrieved from resting THP-1 and MDMs. A comprehensive overview of all relevant peaks and their molecular assignments is provided in Table 1.

### 3.3. THP-1 Macrophages and MDMs Differ in Their Molecular Composition

**Figure 3:**
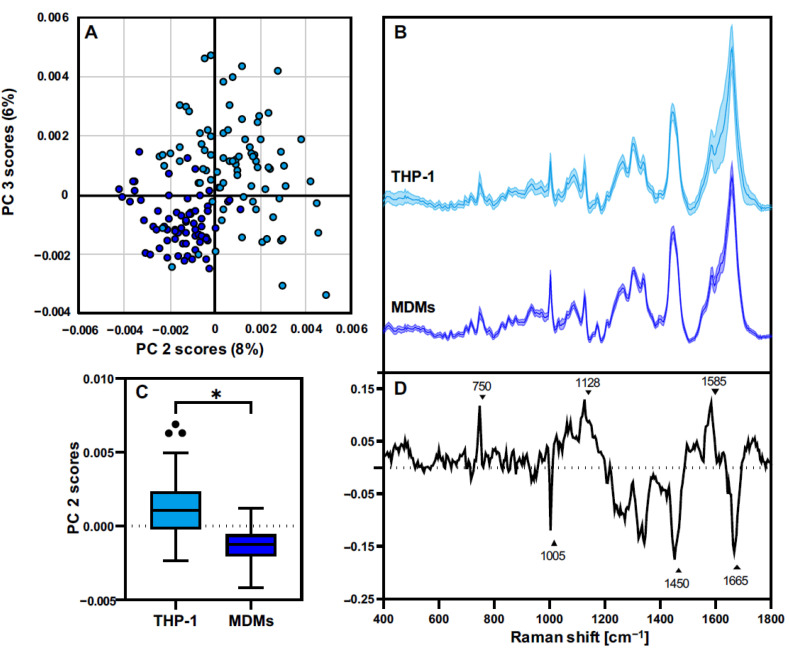
PCA comparison of cytoplasm features identifies differences in the molecular composition between resting THP-1 and MDMs. (**A**) PC 2/PC 3 scores plot of single-cell cytoplasm spectra extracted from THP-1 (light blue) and MDMs (blue) demonstrates a separation of both groups. (**B**) Mean cytoplasm spectra from THP-1 (light blue) and MDMs (blue). (**C**) Statistical analysis of the PC 2 score values reveals significant differences within the cytoplasm from both cell sources. Boxplots show mean single cell PC 2 score values ± SD, *n* = 90, student’s *t*-test, * *p* ≤ 0.05. (**D**) The corresponding PC 2 loadings plot exhibits the molecular vibrations differentiating the cytoplasmic composition between both cell types.

In addition to defining the spatial distribution of subcellular structures, in-depth analyses were performed to investigate the molecular composition in both cell types. Multivariate data analysis allowed us to identify minor spectral differences, as the average spectra exhibited very similar signatures (Figure 3B). For each single-cell Raman scan, spectra were extracted for each of the four cellular components, further used for PCA. First, THP-1 and MDMs were compared in their resting state (M0). PCA comparison of the cytoplasm signatures of non-activated THP-1 and MDMs is demonstrated in Figure 3; PCA results of the other components—nuclei, Lipids A, and Lipids B—are shown in Supplementary Figure S1. The PC-2/PC-3 scores plot exhibits a cluster formation separating THP-1 and MDMs according to the underlying spectral signatures of the cells (Figure 3A). The explained variance represented by PC-2 was significantly different between the two analyzed cell populations (Figure 3C). The corresponding vector highlighting the most influencing spectral features is shown in the loadings plot (Figure 3D). Peaks in the positive PC-2 loadings dominated the spectral information originating from the data clustering in the positive PC-2 scores range—in this case the THP-1 data. Negative peaks in the loadings were more prominent in the spectra from MDMs. The spectral differences between THP-1 and MDMs were assigned to an increased cytochrome c signal at 750, 1128, and 1585 cm^−1^ in THP-1, and a stronger contribution of protein signals at 1003, 1335, 1450, and 1665 cm^−1^ in MDMs. 

### 3.4. THP-1 and MDMs Have a Different Response to Proinflammatory Activation

**Figure 4:**
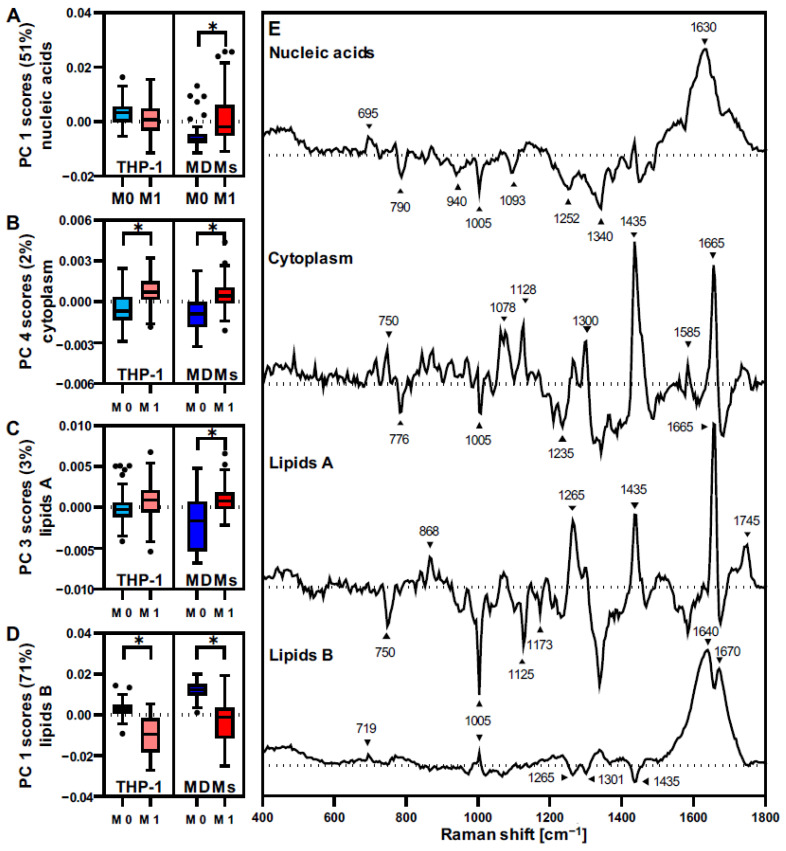
Raman spectra and PCA reflect proinflammatory activation in macrophages. Individual PCAs for (**A**) nucleic acids, (**B**) cytoplasm, (**C**) lipids A, and (**D**) lipids B were performed for data generated for THP-1 and MDMs before and after LPS-INFγ activation. Statistical analyses of the most relevant PC score values demonstrate significant differences in resting and activated MDMs for each of the components, but for THP-1 only within cytoplasmic proteins and lipids B. Boxplots indicate mean single cell PC score values ± SD, *n* = 90, pairwise comparison by student’s *t*-test, * *p* ≤ 0.05. (**E**) Corresponding loadings of the selected PCs describe the molecular changes specific to the separation upon activation.

The effect of macrophage activation was determined and compared between THP-1 and MDMs. The LPS-IFNγ-induced proinflammatory response was evaluated by PCAs, including spectra from THP-1 and MDMs in both resting and activated states (Figure 4, for PCA scores plots see Supplementary Figure S2). Separate analyses were conducted for each of the four cellular structures—nucleic acids, proteins, *lipids A,* and *lipids B*. For all PCAs, the most relevant PCs for separating M0 and M1 macrophages were identified, and single-cell PC score values were statistically compared (Figure 4A–D). Activation-induced changes were especially prominent in nuclei-derived information and *lipids B*, which demonstrated a clustering between resting and activated phenotypes at high explained variance values in PC-1. Differences in cytoplasmic proteins and *lipids A* did only appear at lower PCs. Moreover, MDMs showed significant differences between M0 and M1 states within all analyzed cellular components, whereas proinflammatory changes in THP-1 were mainly linked to alterations in only two of the four components—proteins and *lipids B*. The most influencing Raman bands for each analysis are highlighted in the assigned loadings plots (Figure 4E).

**Figure 5:**
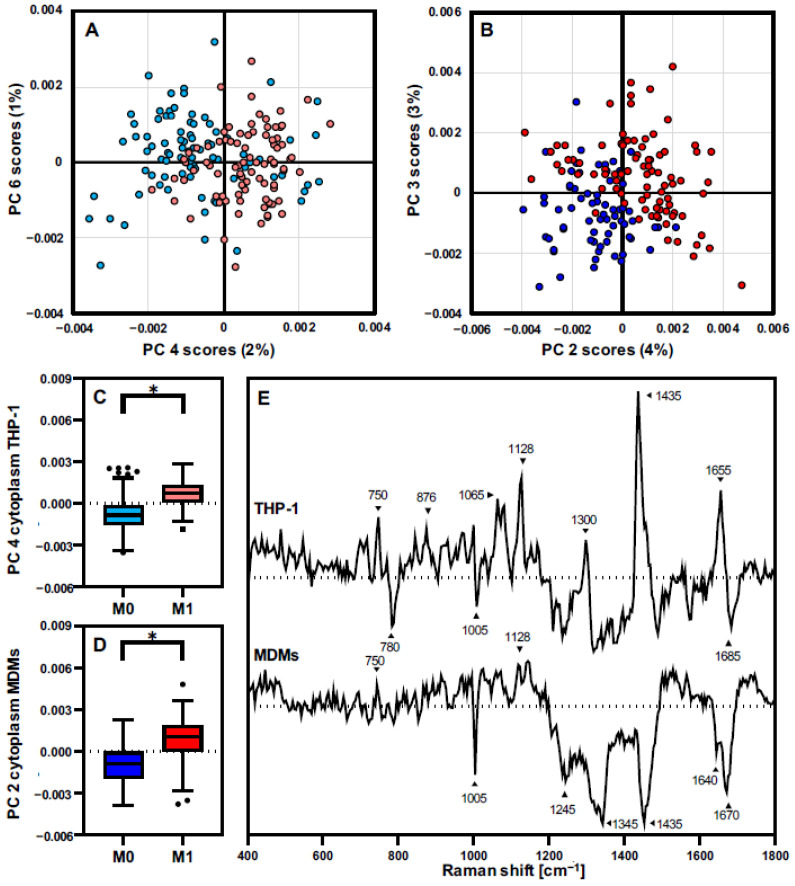
In-depth analysis of cell type-specific changes in cytoplasmic composition upon proinflammatory response. Individual PCAs for the cytoplasmic spectra from THP-1 (**A**,**C**) and MDMs (**B**,**D**) identified the most prominent molecular modifications after LPS-IFNγ treatment for each cell source. Boxplots indicate mean single cell PC score values ± SD, *n* = 90, pairwise comparison by student’s *t*-test, * *p* ≤ 0.05. (**E**) The underlying molecular changes exhibit different signatures and patterns, as indicated by the loadings plots.

To have a deeper insight into the molecular protein modifications driven by the LPS-IFNγ proinflammatory response, independent PCAs including only the data from one cell type were performed. MDMs or THP-1 were separately analyzed with a PCA from extracted spectra from cytoplasm before and after LPS-IFNγ activation (Figure 5). PCA score graphs for THP-1 (Figure 5A) and MDMs (Figure 5B) demonstrated the strongest separation between M0 and M1 macrophages. THP-1 clustering was described by PC 4 with an explained variance of 2%, and MDMs showed a separation in PC 2 with 4% of the total variance. Statistical analysis from the selected PCs scores confirmed that the separation seen on the score graphs was significant (Figure 5C,D). The respective loadings plots for both PCAs (Figure 5E) showed differences in the cytoplasmic molecular composition induced by LPS-INFγ activation for THP-1 and MDMs. However, the separation and assigned molecular changes were driven by different spectral patterns for the two groups. 

## 4. Discussion

The impact of LPS-IFNγ activation on macrophages derived from the THP-1 cell line and primary-isolated PBMCs were compared by assessing their phenotype using RM and Raman imaging, which allowed a holistic view of the cellular composition.

The biological differences between THP-1 macrophages and MDMs have been addressed and discussed by many studies. For example, NFkB activation and gene transcription profiles of THP-1-derived macrophages were similar to that of human MDMs [31,32], whereas opposing results were observed for cytokine expression profiles. Recently, Shiratori et al. showed that polarization and activation profiles differ between MDMs and THP-1 derived macrophages in vitro [33].

In addition, several studies have investigated if macrophage activation can be detected using Raman spectroscopy [34,35]. However, no available study directly compares molecular Raman fingerprints of different macrophage model systems before and after activation.

We were able to show that the information obtained by RM and Raman imaging can be utilized to track molecular changes during the activation process of macrophages and provide insight into diverging molecular mechanisms between different model systems.

To further highlight the benefits of RM, we additionally performed routine FC measurements of common immune surface antigens indicative of immune cell activation.

Although comparable relative responses were observed investigating THP-1 and MDM by FC, differences in baseline expression and relative change in expression after activation were identified, especially for the markers MCP-1 and the costimulatory surface antigen CD86—also known as B7.2, which is essential for successful T cell activation. While a significant increase occurred in MDMs, no increase above resting level was observed in THP-1 macrophages for CD86. As THP-1 is a monocytic leukemia-derived cell line, it can be speculated that the failure to upregulate CD86 for further T cell activation could be an immune evasion mechanism typical for some types of cancer [36]. Similarly, a significant drop of CD163, a scavenger receptor often used as an anti-inflammatory marker as it indicates the clearance of cellular debris and onset of the healing process, was observed in activated MDMs but not in THP-1 macrophages. For MCP-1, opposing effects between the two cultures were identified after activation, with an upregulation in THP-1 macrophages and a downregulation in MDMs. Similar to what was seen for HLA-DR and IL-10, THP-1 cells responded with higher MCP-1 expression when compared with PBMC-derived macrophages [33]. Besides the variation in cell sensitivity, other effects such as the higher genetic variability of MDMs could also contribute to the reported results. In addition, the kinetics of cellular mechanisms can be altered between cell lines and primary cells, with variances in expression profiles growing with incubation time [33]. Altogether, the FC results suggest that the THP-1 cell line lacks some important mechanisms of proinflammatory actions, such as the up- or downregulation of immune response-modulating antigens that are still present in MDMs.

While the FC analysis of surface marker antigens and intracellular cytokines provides a first assessment of activated signaling pathways, it does not capture the extended effects of cellular remodeling. Previous studies have reported a less pronounced polarization profile of THP-1 macrophages after proinflammatory activation compared to MDMs [18]. Here, we were able to show that THP-1 macrophages and MDMs both responded to the challenge with LPS/IFNy; however, analysis of the Raman spectra revealed distinct remodeling patterns in individual cell components.

RM has been widely employed for the phenotypic classification of cells [13,15,37]. Nevertheless, the characterization and identification of macrophage polarization or activation via RM remains in its early stages. Pavillon [34] and Töpfer et al. [35] have used RM to characterize the monocyte cell lines Raw264 and THP-1 to recapitulate the proinflammatory response after LPS-IFNγ treatment. In our study, we demonstrated that besides marker-independent localization of subcellular structures using Raman imaging, RM could identify subtle changes in the molecular composition of nucleic acids, proteins, and lipids that occur upon macrophage inflammatory response in the investigated cell models. Different spectral signatures were obtained from resting THP-1 and MDMs, and cytoplasm-derived PCA scores plots showed a highly heterogeneous scattering in the data. Major differences within the spectral signatures were described by increased cytochrome c bands at 750, 1128, and 1585 cm^−1^ in THP-1 spectra, assigned to the activity of mitochondria, which are known to be regulatory organelles that highly contribute to the immune response [38]. Moreover, differences in cytoplasm composition could correspond to the observed tendency towards increased levels in basal cytokine expression of THP-1 macrophages demonstrated by FC data. MDM spectra showed a stronger impact of protein and amino acid features, demonstrated by increased bands at 935 cm^−1^ (C-C stretching; proline, valine) [39,40], 1449 cm^−1^ (C-H vibration) [41], and 1665 cm^−1^ (Amide I) [42], which are commonly associated with structural vibrations related to the 3D configuration of proteins but can also correlate to a spectral contribution of the inflammasome, a multiprotein complex involved in the initiation of an immune response [43]. Furthermore, the other analyzed cellular structures (nuclei and lipids) demonstrated differences in their molecular composition between the two cell sources (Figure S1). These findings of individual characteristics of resting cell line-derived and primary macrophages comply with previous studies reporting differences in resistance to apoptosis, number of mitochondria, and cytokine expression between THP-1-derived macrophages and MDMs, which are also in dependence on the used THP-1 differentiation protocol [16,18].

In addition to resting macrophages, the sensitivity to LPS-IFNγ-driven proinflammatory activation was monitored and appeared to have greater effects on the phenotypic remodeling of MDM compared to THP-1. MDM activation resulted in significant modifications of nucleic acids, proteins, *lipids A,* and *lipids B*. By contrast, THP-1 activation was only significantly reflected in protein and *lipids B* composition. Based on our PCA results, proteins and *lipids B* are the cellular components that are more sensitive and robust to indicate macrophage polarization. An in-depth analysis of the loadings plots demonstrated a decrease in DNA contribution, increased lipid-related spectral features, and shifts in the protein patterns upon activation. Protein spectral changes due to macrophage activation were dominated by changes in structural bands at 1435 cm^−1^ (CH_2_ scissoring) [44] and 1665 cm^−1^ (Amide I) [42]. The modifications in *lipids B* reflect a prominent amide I with a modified topography at the sub-bands center at 1640 cm^−1^ and 1670 cm^−1^. Alterations within the amide I substructure upon macrophage polarization have been linked to changes in lipid composition [45,46], more specifically to the composition of phospholipids [47]. THP-1 and MDMs showed similar trends regarding these major features; however, relative changes appeared to be more significant for MDMs. Comparable results were obtained by Chaudary et al., who reported different spectral patterns and changes occurring for THP-1 and primary blood-derived cells upon monocyte-to-macrophage differentiation [48]. However, the authors applied different activation protocols among the cells—using PMA for THP-1 and LPS for MDMs. This might affect the polarization pathway and efficacy. THP-1-derived macrophages have been reported to express lower levels of CD14 when compared with MDMs [49]. This surface protein plays an important role in detecting LPS as it forms a highly sensitive complex with TLR4 and MD2 [50]. Accordingly, MDMs have been reported to be more responsive to LPS-activation than THP-1 monocytes [50], which is also reflected by our Raman data, showing relatively higher effects upon activation in MDMs compared to THP-1 cells. These results emphasize the necessity to carefully select an appropriate cell source for an in vitro model.

## 5. Conclusions

Our study shows that RM and Raman imaging allow for the marker-independent evaluation of macrophage LPS-IFNγ-driven proinflammatory changes in both THP-1 and MDMs. Moreover, based on the Raman spectral signature of resting and activated macrophages, it was possible to show that, although both cell types were polarized by the LPS-IFNγ activation, THP-1 cells were less responsive than MDMs. Compared to cell line-derived macrophages, proinflammatory changes in MDMs were reflected in all the identified subcellular structures, suggesting a global response across the immune cell. 

## Figures and Tables

**Table 1:** Major Raman peaks and their molecular assignments.

Wavenumber [cm^−1^]	Vibration Mode	Assignment
**695**	DNA bases (G & T)	DNA [22,23]
**719**	Symmetric stretch vibration of choline group	Phospholipids [24]
**750**	Pyrrole ring breathing	Cytochrome C [25,26]
**776–780**	Symmetric breathing of tryptophan	Proteins [27,28]
**785–790**	Ring breathing modes (DNA/RNA bases)	DNA [22,23]
**868**	C-O-O skeletal vibration	Lipids [24]
**876**	Asymmetric vibration choline N(CH_3_)_3_	Phospholipids [24]
**898**	Adenine	DNA [22,23]
**940**	C-C skeletal vibration (backbone)	Proteins [27,28]
**1005**	Symmetric ring breathing of phenylalanine	Proteins [27,28]
**1093**	Symmetric PO_2_^−^ stretching vibration of the DNA backbone	DNA [22,23]
**1128**	C-N stretching (proteins); C-C vibration in fatty acids	Proteins; Lipids [24,27,28]
**1173**	C-C vibrations fatty acids	Lipids [24]
**1208**	Adenine, Thymine (ring breathing modes)	DNA [22,23]
**1252**	Guanine, cytosine (NH_2_)	DNA [22,23]
**1265**	Amide III; =CH_2_ vibration in lipids	Proteins; Lipids [24,27,28]
**1301**	C-H vibration	(Phospho-) Lipids [24]
**1340**	Adenine, guanine & CH deformation in proteins	DNA [22,23], Proteins [29]
**1435–1445**	CH_3_/CH_2_ scissoring	Lipids [24]
**1450**	CH_2_ deformation	Proteins [27,28]
**1585**	C=C olefinic stretch	Proteins [27,28]
**1630**	DNA bases (C, G, T)	DNA [30]
**1640**	C=C vibrations (fatty acids)	Lipids [24]
**1655–1670**	Amide I, C=C vibrations	Lipids; Proteins [24,27,28]
**1745**	C=O vibrations triacylglycerids	Lipids [24]

## Data Availability

Not applicable.

## Supplementary Materials

The following supporting information can be downloaded at: https://www.mdpi.com/article/10.3390/biomedicines10050989/s1, Figure S1: Principal component analysis (PCA) comparison of extractedspectra from TCA maps of resting THP-1 and MDM-derived macrophages; Figure S2: PCA scores plots corresponding to the multivariate analysis between resting and activated THP-1 and MDM-derived macrophages.
