# Supplementary material for: Raman Microspectroscopy Identifies Biochemical Activation Fingerprints in THP-1- and PBMC-Derived Macrophages
Source: Biomedicines. 2022 Apr 25;10(5):989. doi: 10.3390/biomedicines10050989 (PMC9139061; doi:10.3390/biomedicines10050989)
Supplement: Supplementary file 1 [file biomedicines-10-00989-s001.zip › biomedicines-1654978-supplementary.pdf]

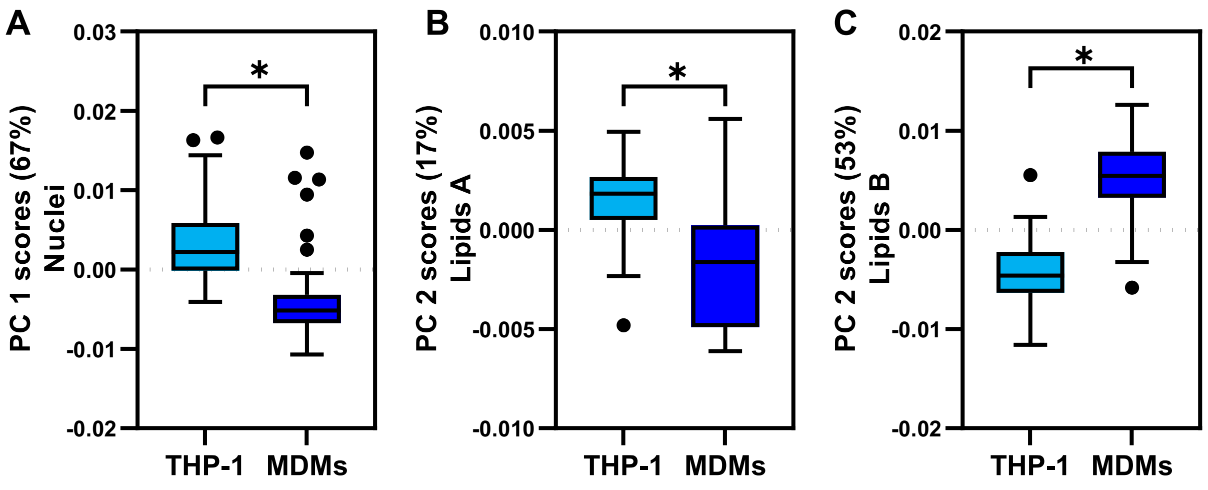

Supplementary Figure S1: Principal component analysis (PCA) comparison of extracted spectra from TCA maps of resting THP-1 and MDM-derived macrophages. Box plots of relevant PC score values for (A) nucleic acids, (B) lipids A and (C) lipids B show a separation of spectral signatures in all selected subcellular structures. n=90 cells per group from 3 independent experiments, t-test, *p<0.05.

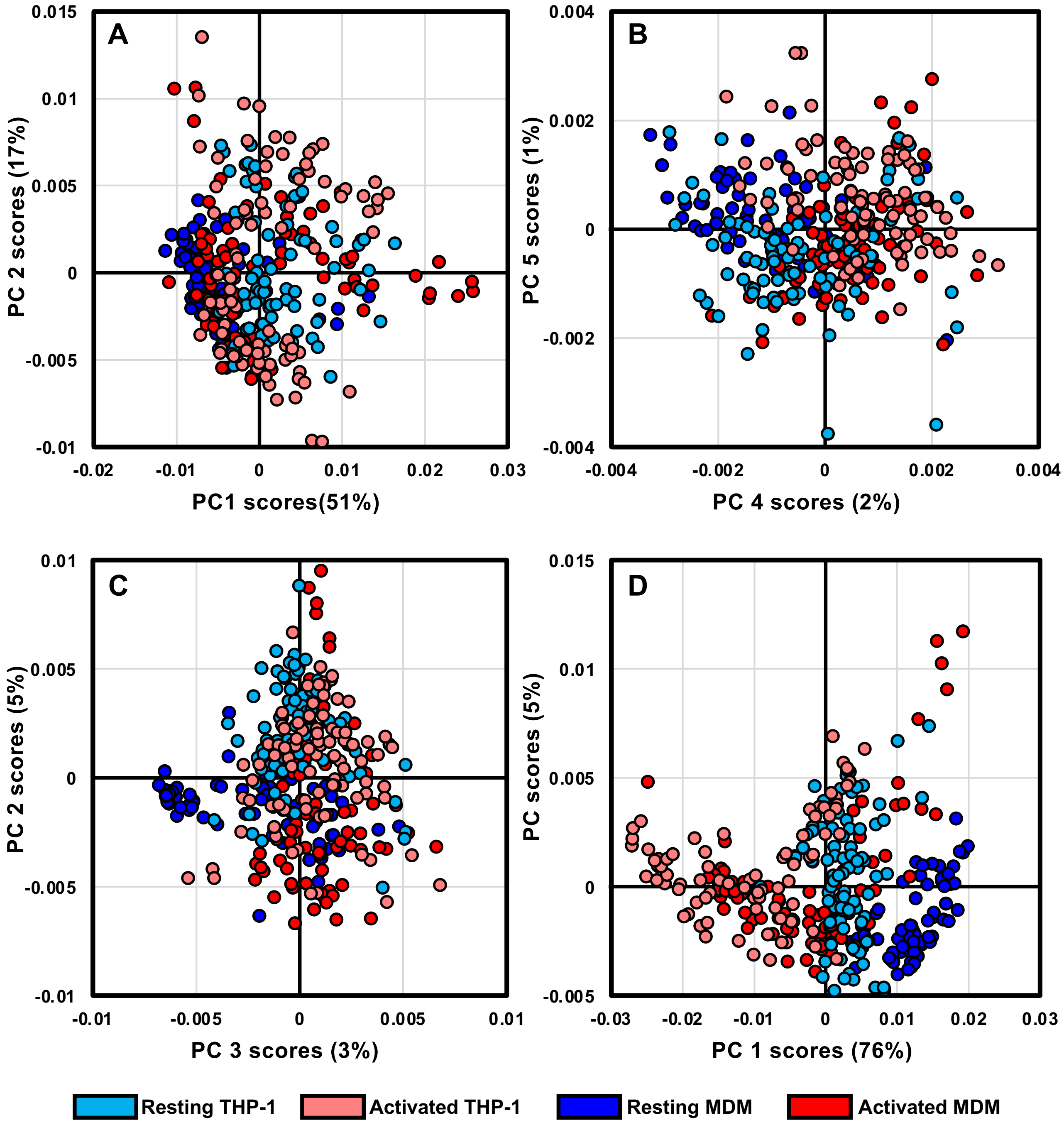

Supplementary Figure S2: PCA scores plots corresponding to the multivariate analysis between resting and activated THP-1 and MDM-derived macrophages (shown in Figure 4) for (A) nuclei, (B) cytoplasm, (C) lipids A and (D) lipids B. n=90 cells per group from 3 independent experiments.
